# Sciatic nerve stimulation alleviates acute neuropathic pain via modulation of neuroinflammation and descending pain inhibition in a rodent model

**DOI:** 10.1186/s12974-022-02513-y

**Published:** 2022-06-15

**Authors:** Chia-En Wong, Chia-Ying Hu, Po-Hsuan Lee, Chi-Chen Huang, Han-Wei Huang, Chih-Yuan Huang, Hsin-Tien Lo, Wentai Liu, Jung-Shun Lee

**Affiliations:** 1 Division of Neurosurgery, Department of Surgery, National Cheng Kung University Hospital, College of Medicine, National Cheng Kung University, Tainan, Taiwan; 2 Department of Cell Biology and Anatomy, Institute of Basic Medical Sciences, College of Medicine, National Cheng Kung University, Tainan, Taiwan; 3 Department of Neurology, National Cheng Kung University Hospital, College of Medicine, National Cheng Kung University, Tainan, Taiwan; 4 Department of Bioengineering, University of California, Los Angeles, Los Angeles, CA 90095 USA; 5 California NanoSystems Institute, University of California, Los Angeles, Los Angeles, CA 90095 USA; 6 Department of Electrical and Computer Engineering, Los Angeles, Los Angeles, CA 900095 USA; 7 Brain Research Institute, University of California, Los Angeles, Los Angeles, CA 900095 USA; 8 Institute of Basic Medical Sciences, College of Medicine, National Cheng Kung University, Tainan, Taiwan; 9 Department of Neurosurgery, National Cheng Kung University Hospital, No. 138, Sheng-Li Road, Tainan, 70428 Taiwan

**Keywords:** Sciatic nerve stimulation, Peripheral nerve stimulation, Neuropathic pain, Nerve root ligation, Neuroinflammation, Descending pain inhibition

## Abstract

**Background:**

Neuropathic pain (NP) is characterized by abnormal activation of pain conducting pathways and manifests as mechanical allodynia and thermal hypersensitivity. Peripheral nerve stimulation is used for treatment of medically refractory chronic NP and has been shown to reduce neuroinflammation. However, whether sciatic nerve stimulation (SNS) is of therapeutic benefit to NP remains unclear. Moreover, the optimal frequency for SNS is unknown. To address this research gap, we investigated the effect of SNS in an acute NP rodent model.

**Methods:**

Rats with right L5 nerve root ligation (NRL) or Sham surgery were used. Ipsilateral SNS was performed at 2 Hz, 20 Hz, and 60 Hz frequencies. Behavioral tests were performed to assess pain and thermal hypersensitivity before and after NRL and SNS. Expression of inflammatory proteins in the L5 spinal cord and the immunohistochemical alterations of spinal cord astrocytes and microglia were examined on post-injury day 7 (PID7) following NRL and SNS. The involvement of the descending pain modulatory pathway was also investigated.

**Results:**

Following NRL, the rats showed a decreased pain threshold and latency on the von Frey and Hargreaves tests. The immunofluorescence results indicated hyperactivation of superficial spinal cord dorsal horn (SCDH) neurons. Both 2-Hz and 20-Hz SNS alleviated pain behavior and hyperactivation of SCDH neurons. On PID7, NRL resulted in elevated expression of spinal cord inflammatory proteins including NF-κB, TNF-α, IL-1β, and IL-6, which was mitigated by 2-Hz and 20-Hz SNS. Furthermore, 2-Hz and 20-Hz SNS suppressed the activation of spinal cord astrocytes and microglia following NRL on PID7. Activity of the descending serotoninergic pain modulation pathway showed an increase early on PID1 following 2-Hz and 20-Hz SNS.

**Conclusions:**

Our results support that both 2-Hz and 20-Hz SNS can alleviate NP behaviors and hyperactivation of pain conducting pathways. We showed that SNS regulates neuroinflammation and reduces inflammatory protein expression, astrocytic gliosis, and microglia activation. During the early post-injury period, SNS also facilitates the descending pain modulatory pathway. Taken together, these findings support the therapeutic potential of SNS for acute NP.

**Supplementary Information:**

The online version contains supplementary material available at 10.1186/s12974-022-02513-y.

## Background

Neuropathic pain (NP), defined as pain caused by a primary lesion of the nervous system, is characterized by abnormal activation of pain conducting pathways [1]. Mechanical allodynia and thermal hypersensitivity are commonly observed in patients with NP and animal models of NP, including rats with L5 nerve root ligation (NRL) [2, 3].

Dysregulated pain signaling and modulation in the central nervous system (CNS) play a critical role in NP [1, 4]. The pathogenesis of NP following NRL is characterized by neuroinflammation in the spinal cord dorsal horn (SCDH) at the corresponding level [5, 6]. Injury of the sciatic nerve has been shown to induce proliferation and hypertrophy of spinal cord astrocytes via activation of the mitogen-activated protein kinase signaling pathway [7]. Activation of extracellular signal-regulated kinase in spinal microglia and astrocytes following spinal nerve injury has also been reported [8]. Such changes in spinal cord glial cells are known to facilitate hyperactivation of somatosensory neurons in the SCDH and result in NP [8–10]. Together, these findings suggest a pivotal role of CNS neuroinflammation in the development of acute pain hypersensitivity and the subsequent transition from acute to chronic NP. Thus, modulation of CNS neuroinflammation and the associated hyperactivation of somatosensory pain signaling pathways is a potential treatment strategy for alleviating NP.

Neuromodulatory techniques, such as peripheral nerve stimulation (PNS) and spinal cord stimulation, have been used to treat debilitating pain in patients who fail to respond to or cannot tolerate pharmacological treatments [11]. Emerging clinical evidence supports the clinical efficacy of PNS in the management of chronic pain. Sator-Katzenschlager et al. reported a reduction in pain scores of more than 50% and a decrease in the doses of required analgesics in 111 NP patients receiving PNS [12]. Similar results were obtained in patients with chronic back pain, who had lower pain scores and analgesic consumption after PNS [13, 14]. Goebel et al. successfully utilized PNS to achieve sustained pain relief in an amputated patient with complex regional pain syndrome who had failed to respond to SCS [15]. Despite such evidence of PNS being successfully used to treat chronic pain, its applications in treating acute pain were not reported until recent years [16]. For example, Ilfield et al. applied PNS in the immediate postoperative period following knee surgery and reported an average 85% improvement of pain scores [17].

Previous animal experiments have demonstrated that PNS can increase the pain threshold in NRL rats [18] and shed light on potential mechanisms of PNS, such as gate-control-induced paresthesia [19], inflammatory modulation [20, 21], and endogenous pain inhibition pathways [20]. However, a detailed investigation of the mechanism of PNS is lacking. Furthermore, there is limited research on the frequency used in PNS, which is an imperative parameter affecting the response. PNS at a frequency of 100–10,000 Hz is associated with gate-control-induced paresthesia to achieve analgesia [19, 22], whereas PNS at a lower frequency (2–30 Hz) is reported to have a modulatory effect on inflammation [21, 23]. Thus, further investigation of frequency is essential for achieving optimal pain relief from PNS. Moreover, the existing studies regarding PNS in the treatment of acute pain conditions are limited to nociceptive pain, such as postoperative pain [17, 24]. The efficacy of PNS for acute NP is yet to be investigated.

To address these knowledge gaps in NP research, the present study aimed to elucidate the potential role of sciatic nerve stimulation (SNS) with graded frequency in an acute NP model of NRL rats. Three different electrical stimulation frequencies, namely 2 Hz, 20 Hz, 60 Hz, were compared in terms of their analgesic efficacy and their effects on histomorphology in the SCDH and pain-related regions of the brainstem.

## Methods

### Animals

This study was approved by the Animal Ethics Committee of National Cheng Kung University (NCKU) in Tainan, Taiwan (IACUC approval number: 109195). All surgical interventions, perioperative care, and treatments were performed in accordance with the guidelines of the Institute of Animal Use and Care Committee at NCKU. Adult male Sprague-Dawley rats (250–300 g) were obtained from BioLASCO (Nangang, Taipei, Taiwan) and housed at 25 ± 2 °C under a 12 h light–dark cycle. Food and water were available ad libitum. Efforts were made to reduce the number of animals used and to minimize animal suffering.

### L5 spinal nerve root ligation model

The surgical procedures for L5 nerve root ligation (NRL) were performed as previously described with some modifications [25]. The rats were anesthetized by an intraperitoneal injection of Zoletil® 50 (40 mg/kg; Virbac, Carros, France), administrated with enrofloxacin (5 mg/kg, Bayer, Leverkusen, Germany) and placed in a prone position. In brief, the paraspinal muscles were split via a paramedian incision and gently retracted to expose the L5 transverse process. The L5 transverse process was then removed to expose the underlying L5 nerve root. L5 spinal nerve ligation was performed with a 4–0 non-absorbable suture (Additional file 1: Supplementary figure 1). Wound closure was performed in a layer-by-layer fashion. After NRL, a heating pad was used to maintain the rat’s body temperature at 37 °C until recovery from anesthesia.

### Sciatic nerve electrical stimulation

**Fig. 1:**
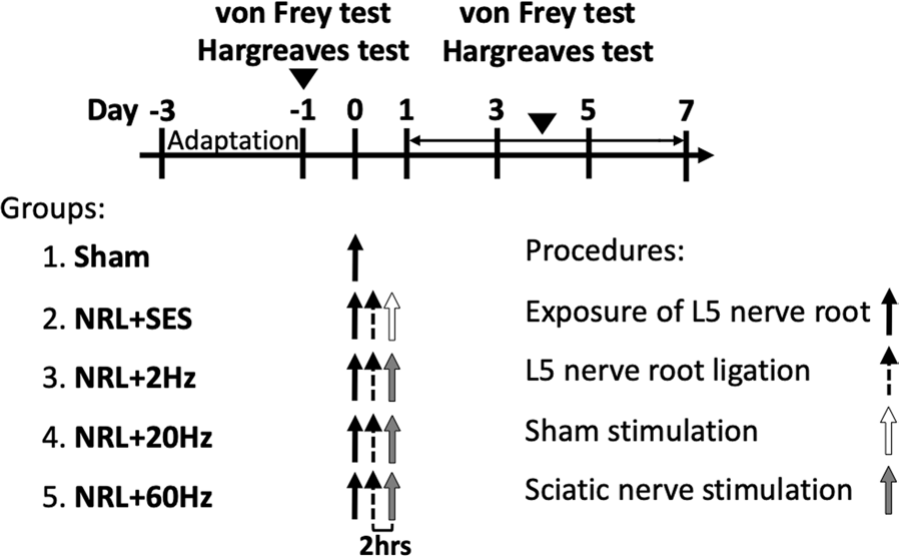
Study protocol for L5 NRL and SNS. The Sham group (group 1) with the L5 nerve root exposed but not ligated was used as the control. In NRL + SES (group 1), the L5 nerve root was exposed and ligated and the SNS electrode was placed distal to the joint of L4, L5, and L6 nerve roots 2 h after NRL but no electrical stimulation was performed. In the SES groups (Groups 3, 4, and 5), NRL and SNS electrode placement was performed as above described and 2-Hz, 20-Hz, and 60-Hz SNS was performed. Behavioral tests, including the von Frey test and Hargreaves test, were performed 1 day before NRL and on PID 1, 3, 5, and 7

Five different sets of animal experiments, including SNS at three frequencies, were performed (Fig. 1): Sham (exposure of L5 nerve root without ligation, *N* = 10); NRL + Sham electrical stimulation (NRL + SES, *N* = 10); NRL + 2 Hz SNS (NRL + 2 Hz, *N* = 10); NRL + 20 Hz SNS (NRL + 20 Hz, *N* = 10); and NRL + 60 Hz SNS (NRL + 60 Hz, *N* = 10). SNS was performed in a 1-h single session starting 2 h after the surgery of L5 NRL. The segment of the stimulated sciatic nerve is ipsilateral to the ligated L5 nerve root at a location distal to the joint of L4, L5, and L6 nerve roots in the thigh. The electric stimulation was delivered via custom-made bipolar electrodes connected to a nerve conduction/electromyography stimulator (ISIS Xpress, inomed Medizintechnik Gmbh, Germany). The delivered stimulus consisted of 2-s-long uniform biphasic pulse trains at a frequency of 2 Hz, 20 Hz, and 60 Hz with 200-μs square wave pulses. The pulse trains were separated by 8-s off intervals. The intensity ranged from 1 to 10 mA. Motor responses were used to confirm the delivery of electric stimulation. The stimulation intensity was subsequently selected as the maximal intensity that did not trigger a motor response. These parameters were adapted from previous studies demonstrating that such stimuli activate all fiber types [26]. In the NRL + SES group, Sham electrodes without electrical current were applied to the sciatic nerve.

### Behavioral tests

Starting three days before surgery, the rats were introduced to the testing environment daily to allow for acclimation. The rats were placed in the environment used for the mechanical and thermal tests for 30 min before the tests were performed. Before and 1, 3, 5, and 7 days after surgery, the plantar surface of both ipsilateral and contralateral hind paws was probed using electrical von Frey tips (BIO-EVF5, Bioseb, France) to measure the thresholds of mechanical touch sensitivity (von Frey test). The tip was gently applied upward onto the rat’s middle plantar surface and force was slowly exerted until the rats withdrew, flicked, or licked their paw. The reading of the largest force (in grams) was automatically recorded. Each rat was tested five times separated by 15-s intervals.

Hargreaves test was performed on preoperative day 1 and 1, 3, 5, and 7 days after surgery to evaluate thermal sensitivity using a Plantar Test Apparatus (Ugo Basile, Comerio, Italy). An infrared heat source (50 W) was adjusted so that naïve rats had withdrawal latencies of 9–12 s. The heat source was focused on the plantar surface of the ipsilateral and contralateral hind paw and the time taken to withdraw from the heat stimulus was recorded. Each hind paw was tested three times separated by intervals of 2 min.

### Western blot analysis

L4–L6 spinal cords were freshly collected and homogenized with T-PER reagent buffer containing protease inhibitor mixture (Thermo Scientific). Homogenates were centrifuged at 12,000 rpm for 15 min at 4 °C and the supernatants were collected as total cell lysates. Proteins extracts (30 μg), which were quantified using a protein assay kit (Micro BCA™, Thermo Fisher Scientific Inc.), were resuspended in loading buffer and subjected to polyacrylamide gel electrophoresis followed by transfer to a nitrocellulose membrane for 2 h. The membranes were blocked with 5% non-fat milk in Tris-buffered saline with Tween-20 (TBST, 20 mM Tris base, 130 mM NaCl, 0.1% Tween-20) and then incubated overnight at 4 °C with the following primary antibodies: anti-α-tubulin (GeneTex GTX112141, 1:10,000), anti- NF-κB (Cell Signaling Technology #8242, 1:2000), anti-IL-1β (Origene TA321162, 1:2000), anti-TNF-α (abcam ab6671, 1:2000), and anti-IL-6 (abcam ab6672, 1:2000). After primary antibody incubation, the membranes were washed with TBST and incubated with horseradish peroxidase-conjugated secondary antibodies (Jackson ImmunoResearch Inc., West Grove, PA, USA) for 1 h at room temperature. Following the final washing step, Western blot analysis was performed using an enhanced chemiluminescence detection kit (WBKLS000, MerckMillipore/Merck KGaA) and visualized using a luminescence imaging system (Azure Biosystems). The protein level was normalized to α-tubulin and analyzed using ImageJ software (NIH, USA). All described experiments were performed in triplicate.

### Immunofluorescence staining

After the rats were anesthetized, transcardial perfusion was performed using ice-cold normal saline followed by 4% paraformaldehyde in 0.1 M phosphate buffer at pH 7.4. Spinal cord tissues were collected and postfixed in 4% paraformaldehyde at 4 °C overnight. Afterward, the spinal cord tissues were dissected into 10 mm segments, centered over the L5 level, immersed in PBS with 30% sucrose for cryoprotection at room temperature for 48 h, and embedded in optimal cutting temperature compound in liquid nitrogen. Transverse cryosections of the spinal cord and brainstem tissues were cut at 20 μm using a cryostat (LEICA CM1950) at − 20 °C. Ten sections at intervals of 480 μm were sampled from each animal for further immunofluorescence analysis.

Prior to immunofluorescence staining, sections were rinsed with 0.01 M phosphate-buffered saline (PBS), permeabilized, blocked with 2% normal goat serum (prepared in PBS supplemented with 0.1% BSA and 0.1% Triton X-100) for 5 min, and blocked with 10% normal goat serum (prepared in PBS supplemented with 0.1% BSA and 0.1% Triton X-100) for 20 min. The sections were then incubated overnight at 4 °C with the following primary antibodies: anti-c-fos (Abcam ab190289, 1:1000), anti-GFAP (Sigma G3893, 1:400), anti-Iba1 (GeneTex GTX635363, 1:200), and anti-TPH2 (Abcam ab211528, 1:500). This was followed by incubation with secondary fluorescent-dye conjugated secondary antibodies (Thermo Fisher Scientific Inc.) at room temperature for 1 h. After rinsing with 0.01 M PBS, the sections were mounted with media with 4′,6-diamidino-2-phenylindole (Abcam, Cambridge, UK). The fluorescent images were captured using a fluorescence microscopy system (Nikon M568E; Minato City, Tokyo, Japan). All described experiments were performed in triplicate.

### Statistical analysis

Continuous variables were expressed as the mean ± standard deviation. Data from two groups were compared using Student’s *t*-test. Data from three or more groups were compared using one-way analysis of variance with Bonferroni’s post hoc test. Differences were considered statistically significant at *p* < 0.05.

## Results

### SNS alleviates pain and thermal hypersensitivity in L5 NRL rats

**Fig. 2:**
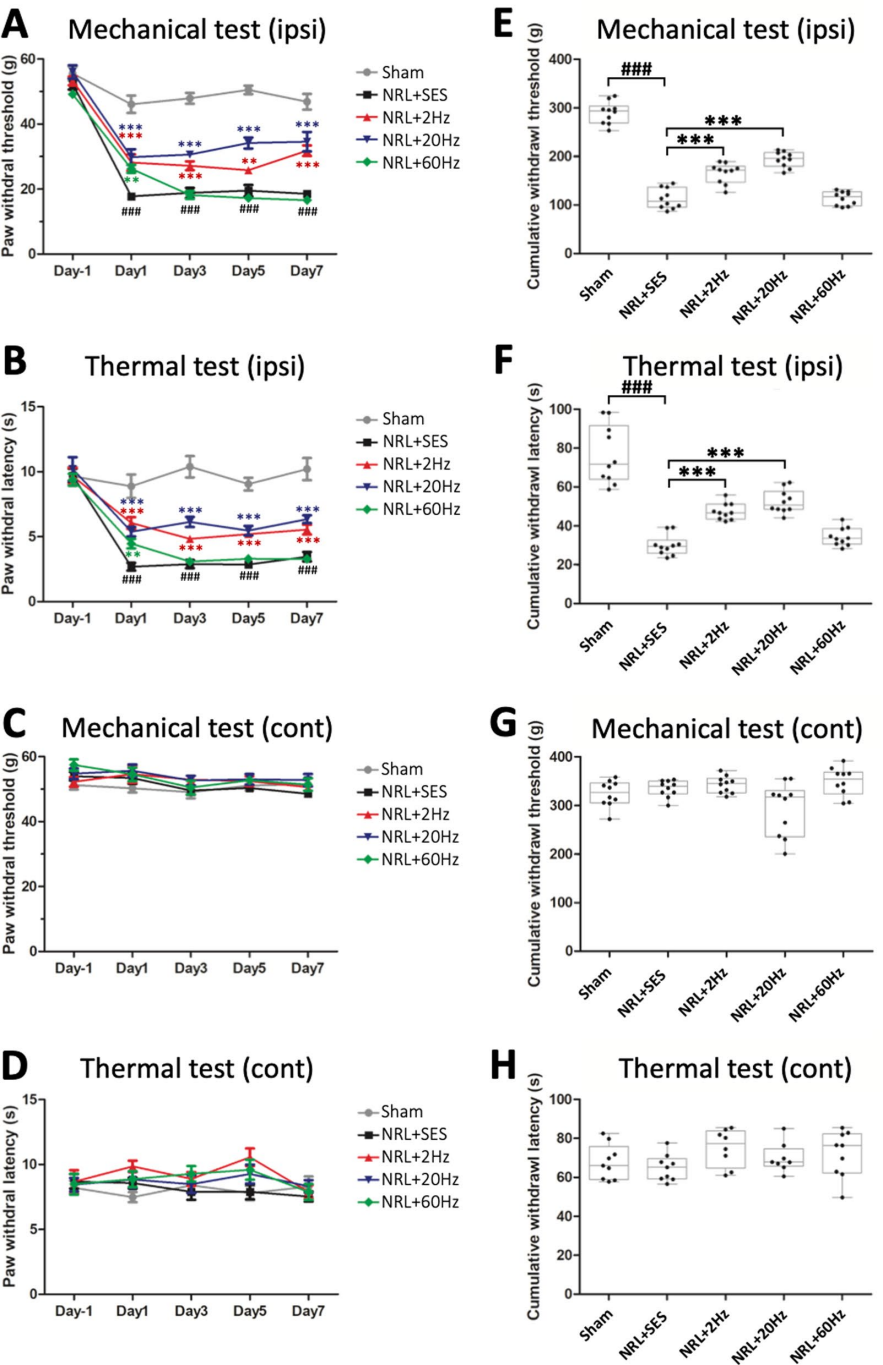
Effects of SNS on pain and thermal hypersensitivity in L5 NRL rats. The paw withdrawal threshold and latency on the ipsilateral (**a**, **b**) and contralateral side (**c**, **d**) to NRL and SNS 1 day before NRL and PID 1, 3, 5, and 7 were recorded (*N* = 10). Data are expressed as mean ± SD. The area under the curve was calculated as the cumulative withdrawal threshold and latency on the ipsilateral (**e**, **f**) and contralateral side (**g**, **h**). ^###^*p* < 0.001 compared to Sham. **p< 0.01 compared to NRL + SES. *p < 0.001 compared to NRL + SES

To evaluate the effect of SNS, we first evaluated mechanical (von Frey test) and thermal (Hargreaves test) pain sensitivity in L5 NRL rats. The Sham group with the L5 root exposed but not ligated was used as a control. Compared to the Sham group, NRL + SES rats exhibited a significantly lower paw withdrawal threshold and decreased paw withdrawal latency from post-injury day 1 (PID1) to PID7 on the ipsilateral side (Fig. 2a, b) but not the contralateral side (Fig. 2c, d). To further investigate the therapeutic potential of SNS and the efficacy of different electrical frequencies, the pain behaviors of SNS rats following 2 Hz, 20 Hz, and 60 Hz stimulation were evaluated. On PID1, all frequencies of SNS increased the paw withdrawal thresholds and latencies compared to NRL + SES. On PID 3, 5, and 7, increased paw withdrawal thresholds and latencies compared to NRL + SES were observed for 2-Hz and 20-Hz SNS, but not 60-Hz SNS (Fig. 2a, b). The pain and thermal sensitivities of the contralateral paw were unchanged (Fig. 2c, d). We then calculated the cumulative withdrawal thresholds and latencies for each rat (Fig. 2e–h). The results showed that L5 NRL resulted in significantly decreased ipsilateral cumulative withdrawal thresholds and latencies and that the decreases were ameliorated by 2-Hz and 20-Hz SNS, but not 60-Hz SNS.

### Hyperactivation of SCDH superficial laminar neurons is normalized following SNS in L5 NRL rats

Based on the pain behavior test results, we wondered if the alterations in pain behavior were correlated with alterations in the neuronal activity of pain transmitting neurons. Therefore, we investigated the neuronal activity of L5 SCDH superficial laminar neurons.

**Fig. 3:**
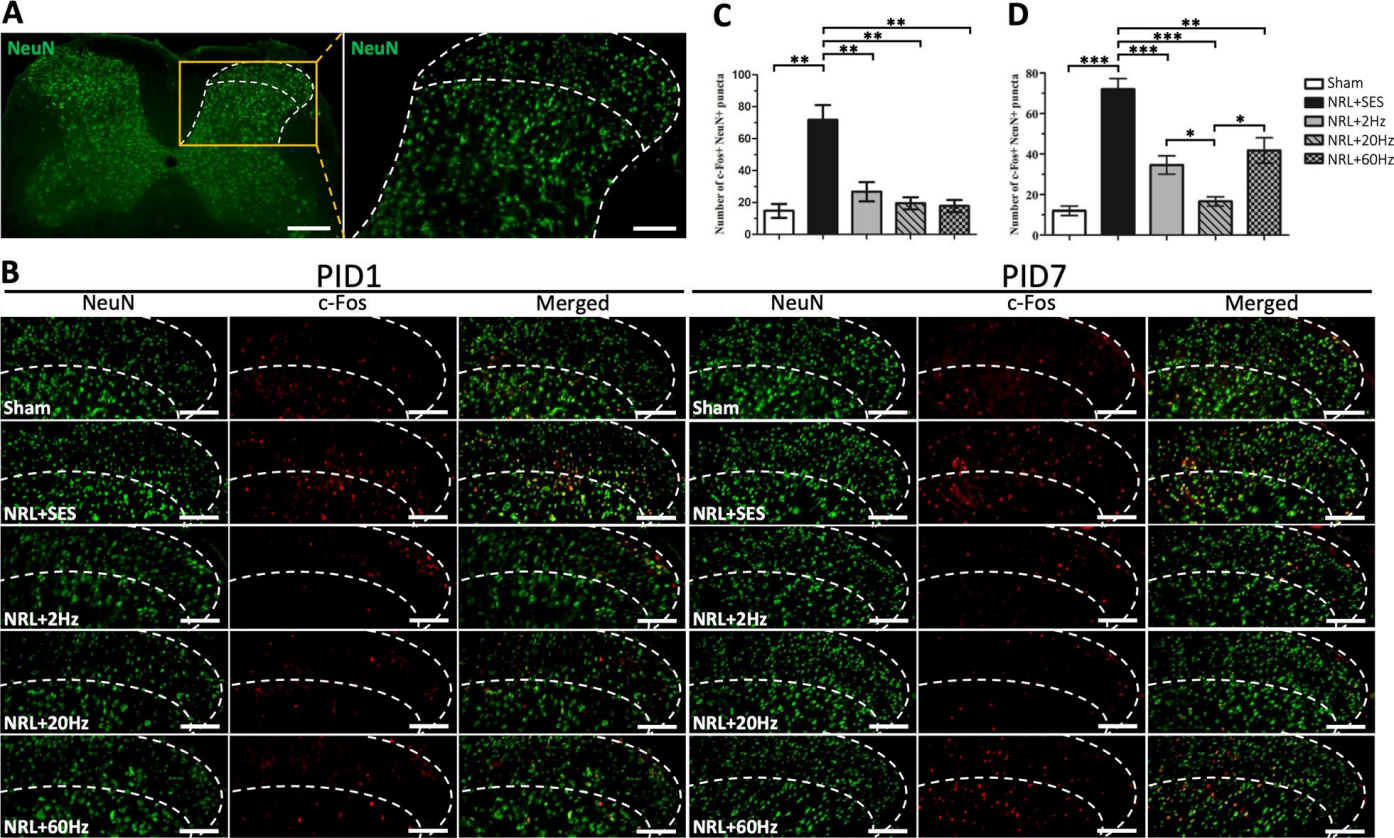
Activity of SCDH superficial laminar neurons following SNS in L5 NRL rats on PID1 and PID7. Transverse sections of the L5 spinal cord were obtained from NRL rats on PID1 (*N* = 4) and PID7 (*N* = 5). The border between the superficial (lamina I–III) and deep lamina was set by the heterogenic neuronal size of lamina IV, compared to the smaller cells of lamina III [27], as indicated by the dashed lines (**a**). Double-immunofluorescence staining of neuronal marker NeuN (green) and c-fos (red) (**b**). Colocalization of NeuN and c-fos appears yellow in the merged image. Quantification of the number of NeuN/c-fos co-localized cells in a single SCDH section on PID1 (**c**) and PID7 (**d**). Scale bar = 200 μm. Data are expressed as mean ± SD. *p < 0.05, **p < 0.01, ***p < 0.001

Figure 3 presents the immunofluorescence images of frozen SCDH sections on PID1 and PID7. The border between the superficial and the deep laminae (dashed line) was established based on the difference in the heterogenic neuronal size characteristic of lamina IV versus the smaller cells characteristic of lamina III [27] (see magnification on the right in Fig. 3a). Co-staining of NeuN and c-fos is shown in Fig. 3b. Quantification of c-fos/NeuN co-localized neurons in the L5 SCDH superficial laminar revealed a 4.8 and 5.9-fold increase in the number of activated neurons on PID1 and PID7, respectively, in the NRL + SES group compared with the Sham group (Fig. 3c, d). On PID1, the numbers of activated superficial laminar neurons observed in 2 Hz, 20 Hz, and 60 Hz SNS groups were 62.7%, 72.8%, and 75.3% less, respectively, compared with NRL + SES group. Whereas on PID7, the numbers of activated superficial laminar neurons were 51.8%, 76.9%, and 42.0% less in 2 Hz, 20 Hz, and 60 Hz SNS groups, respectively, compared to NRL + SES. Comparison of the different SNS frequencies showed that 20-Hz SNS resulted in a greater decrease in the SCDH c-fos signal compared to 2 Hz and 60 Hz on PID7. In contrast, there was no difference among the results of 2-Hz, 20-Hz and 60-Hz SNS on PID1.

### Modulatory effect of SNS on expression of spinal cord NF-κB, TNF-α, IL-1β, and IL-6 in L5 NRL rats

Previous studies have shown that neuroinflammation in the SCDH plays an essential role in the formation of NP in NRL animal models [6, 28]. Therefore, we investigated whether SNS impacted the inflammatory response in the SCDH after L5 NRL.

**Fig. 4:**
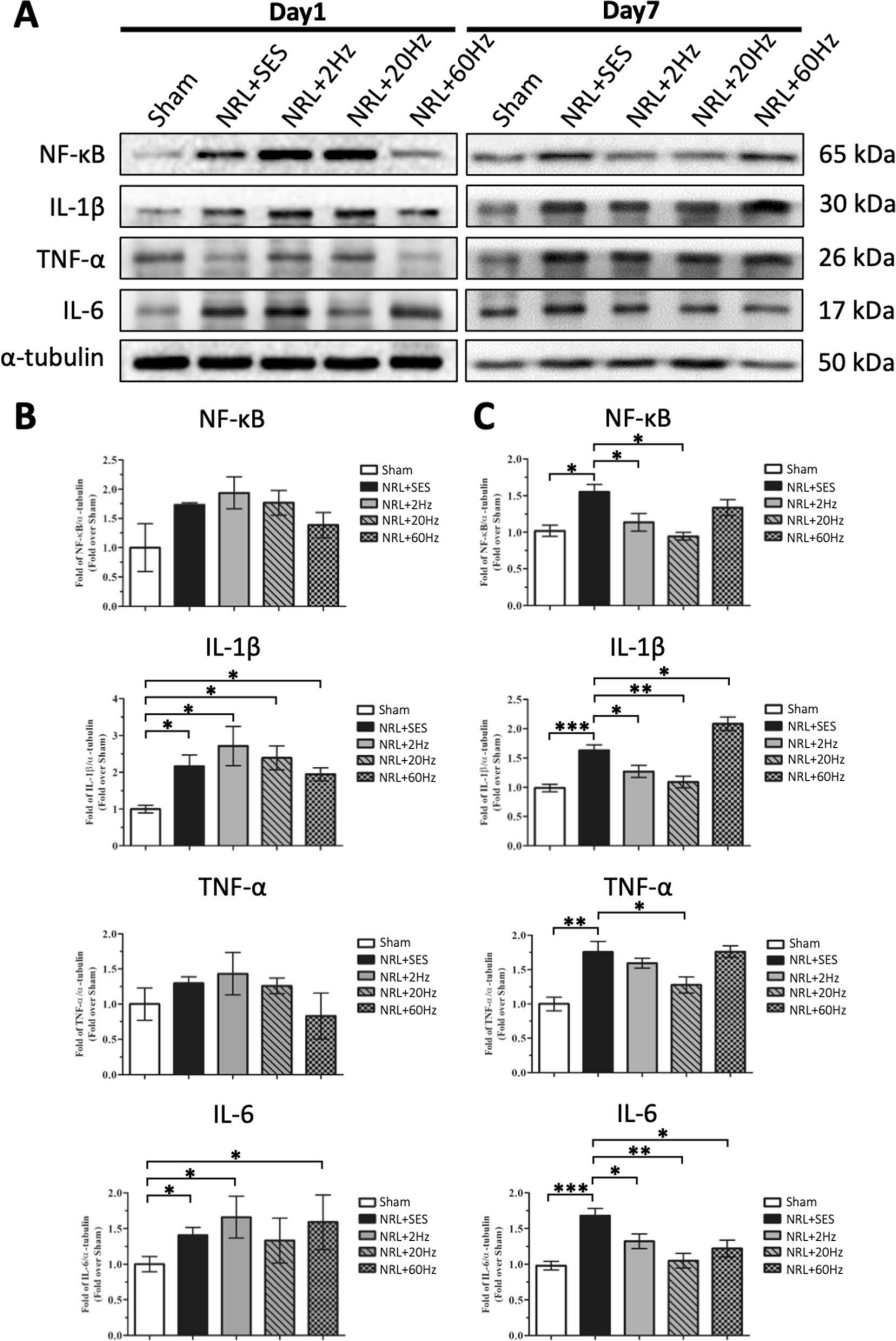
Effect of SNS on the expression of spinal cord NF-κB, TNF-α, IL-1β, and IL-6 in L5 NRL rats. Spinal cord tissues were obtained from NRL rats (N = 5) on PID1 and PID7. Tissue lysates were analyzed by immunoblotting with specific antibodies against NF-κB, TNF-α, IL-1β, and IL-6 (**a**). α-tubulin was used as an internal control. Relative levels of spinal cord NF-κB, TNF-α, IL-1β, and IL-6 on PID1 were quantified by densitometric analysis using ImageJ software (**b**). Relative levels of spinal cord NF-κB, TNF-α, IL-1β, and IL-6 on PID7 were quantified by densitometric analysis using ImageJ software (**c**). Data are expressed as mean ± SD. *p < 0.05, **p < 0.01, ***p < 0.001

Expression of the inflammatory mediator NF-κB and proinflammatory cytokines TNF-α, IL-1β, and IL-6 on PID1 and PID7 were measured by Western blotting (Fig. 4). On PID1, the expression of IL-1β and IL-6 was significantly elevated by 2.2-fold and 1.4-fold, respectively, in the NRL + SES group compared to the Sham group. We also observed a trend of elevated expression of NF-κB and TNF-α on PID1, although it was not statistically significant (Fig. 4b). On PID7, the levels of spinal cord NF-κB, TNF-α, IL-1β, and IL-6 were all significantly elevated by 1.6-fold, 1.8-fold, 1.6-fold, and 1.7-fold in the NRL + SES group compared to the Sham group, respectively (Fig. 4c). On PID1, none of the SNS groups showed significant changes in the expression of spinal cord NF-κB, TNF-α, IL-1β, and IL-6 compared to NRL + SES (Fig. 4b). However, on PID7, we observed that 20-Hz SNS reduced the expression of TNF-α, 2-Hz and 20-Hz SNS reduced the expression of NF-κB, and all frequencies of SNS reduced the expression of IL-1β and IL-6 (Fig. 4c).

### Effects of various SNS frequencies on astrocyte proliferation and microglia activation on PID7 in the SCDH in L5 NRL rats

**Fig. 5:**
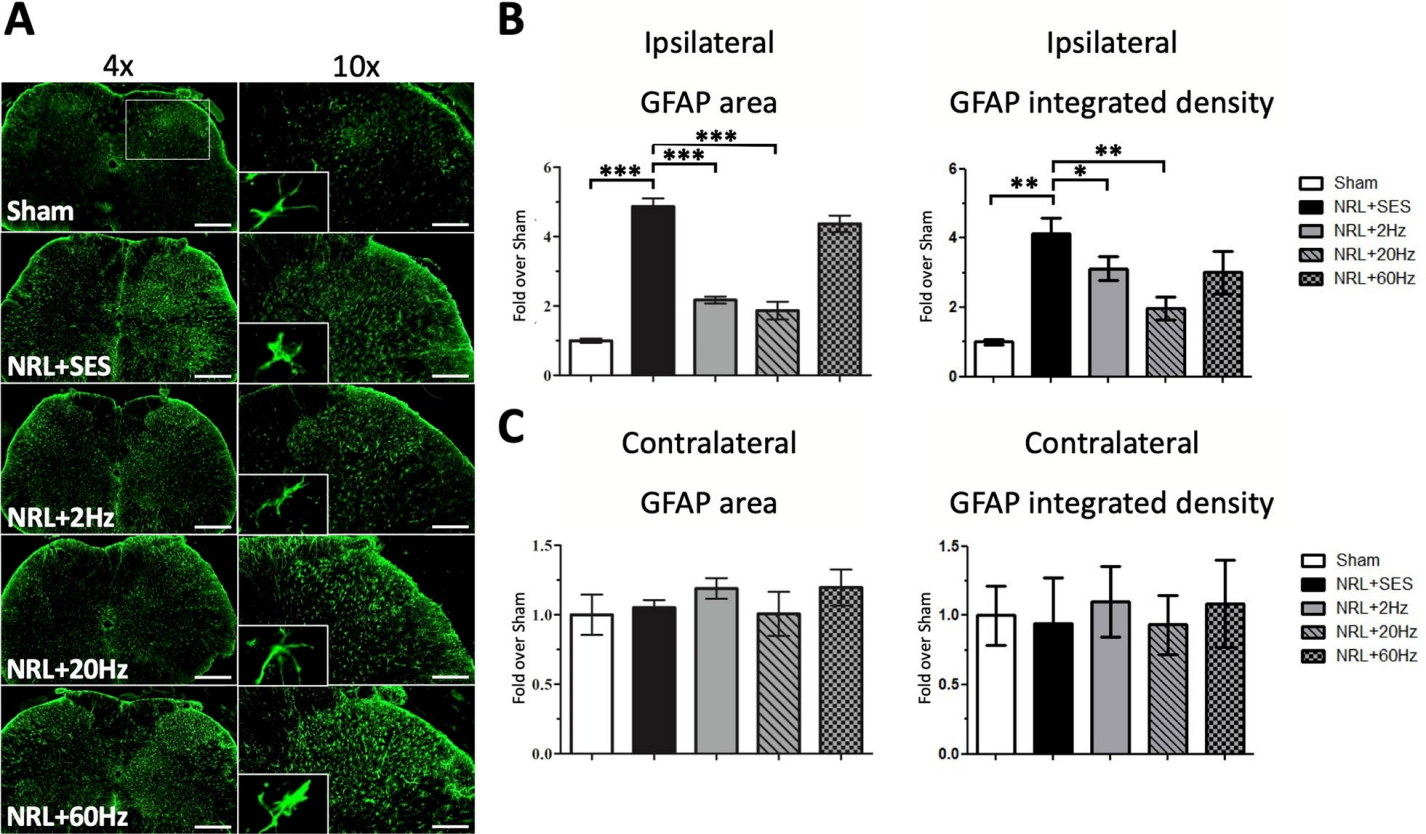
Proliferation of SCDH astrocytes on PID7 following SNS at various frequencies in L5 NRL rats. Transverse sections of L5 spinal cord were obtained from NRL rats on PID7 (*N* = 5). Immunofluorescence staining of the astrocyte marker GFAP (green) on PID7 (**a**). The right panels show magnification (× 10) of the ipsilateral SCDH. The relative area and integrated density of ipsilateral and contralateral GFAP signal was quantified using ImageJ software (**b**, **c**). Scale bars = 500 μm and 200 μm (magnification). Data are expressed as mean ± SD. *p < 0.05, **p < 0.01. ***p < 0.001

Since spinal cord astrocytes and microglia are also involved in neuroinflammation and the formation of NP [29–32], we investigated whether astrocyte proliferation and microglia activation occurred in SCDH in the rat model of acute NP. Figure 5a shows the immunofluorescence staining results of GFAP in frozen SCDH sections on PID7. Quantification of ipsilateral GFAP signal in the L5 spinal cord (Fig. 5a, magnification) revealed a 4.9-fold increase in the GFAP area and a 4.1-fold increase in the GFAP integrated density in the NRL + SES group compared with the Sham group (Fig. 5b). Notably, the increased ipsilateral signal was ameliorated by 2-Hz and 20-Hz SNS, but by not 60-Hz SNS (Fig. 5b). The contralateral GFAP area and integrated density were not affected by L5 NRL and SNS (Fig. 5c).

**Fig. 6:**
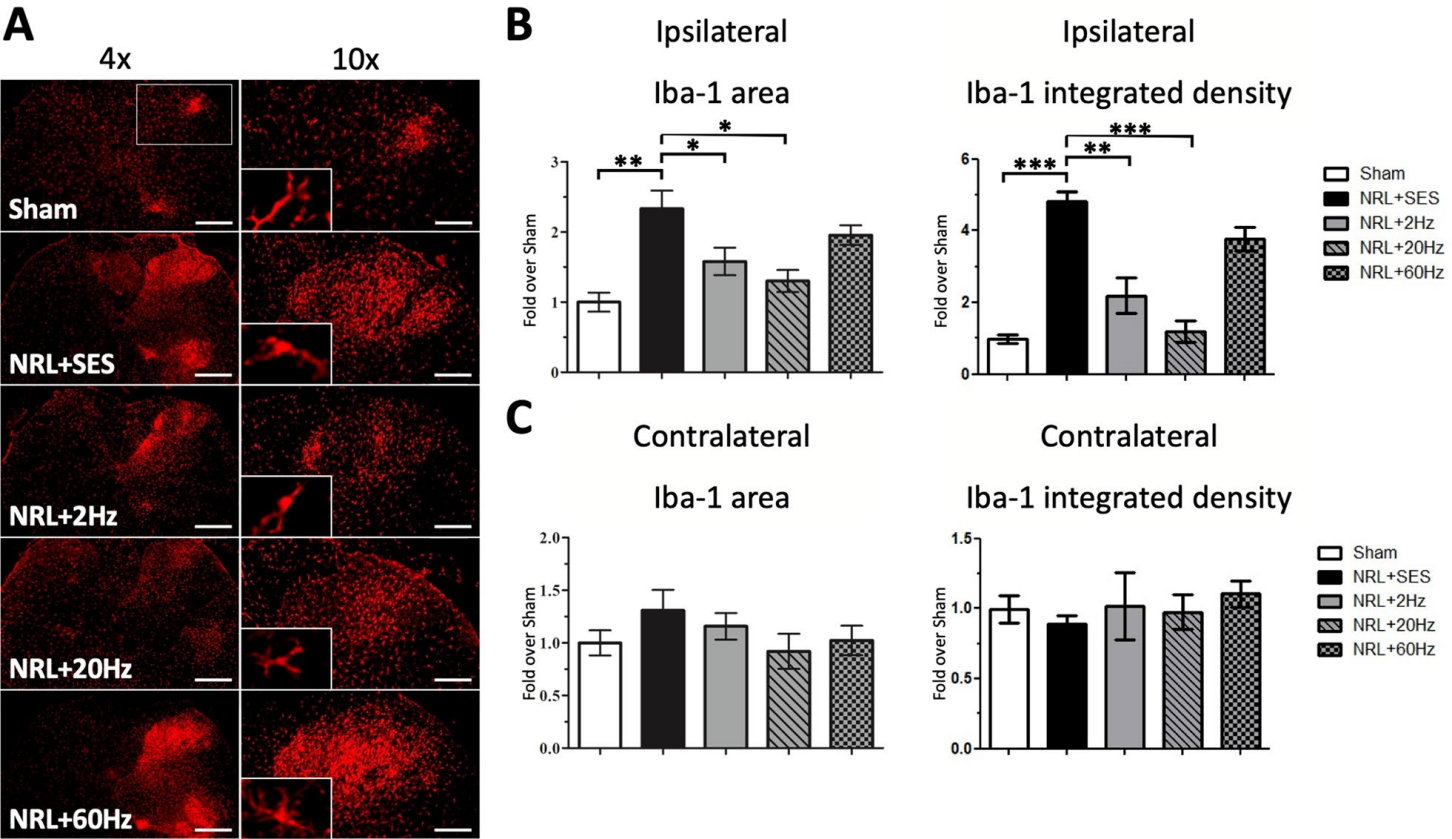
Activation of SCDH microglia on PID7 following SNS at various frequencies in L5 NRL rats. Transverse sections of L5 spinal cord were obtained from NRL rats on PID7 (*N* = 5). Immunofluorescence staining of the microglia marker Iba1 (red) on PID7 (**a**). The right panels show magnification (× 10) of the ipsilateral SCDH. The relative area and integrated density of ipsilateral and contralateral Iba1 signal was quantified using ImageJ software (**b**, **c**). Scale bars = 500 μm and 200 μm (magnification). Data are expressed as mean ± SD. *p < 0.05, **p < 0.01. ***p < 0.001

We also analyzed the immunofluorescence signal for Iba1 in L5 SCDH (Fig. 6a). The results revealed a 2.3-fold increase in the ipsilateral Iba1 area and a 4.8-fold increase in the Iba-1 integrated density in the NRL + SES group compared with the Sham group (Fig. 6b). The increase in the ipsilateral Iba1 signal was mitigated by 2-Hz and 20-Hz SNS, but not 60-Hz SNS (Fig. 6b). On the contralateral side, the difference in the Iba1 area and integrated density were not significant among all groups (Fig. 5c).

### Upregulation of TPH2+ neuronal activity in the rostral ventromedial medulla following SNS in L5 NRL rats

**Fig. 7:**
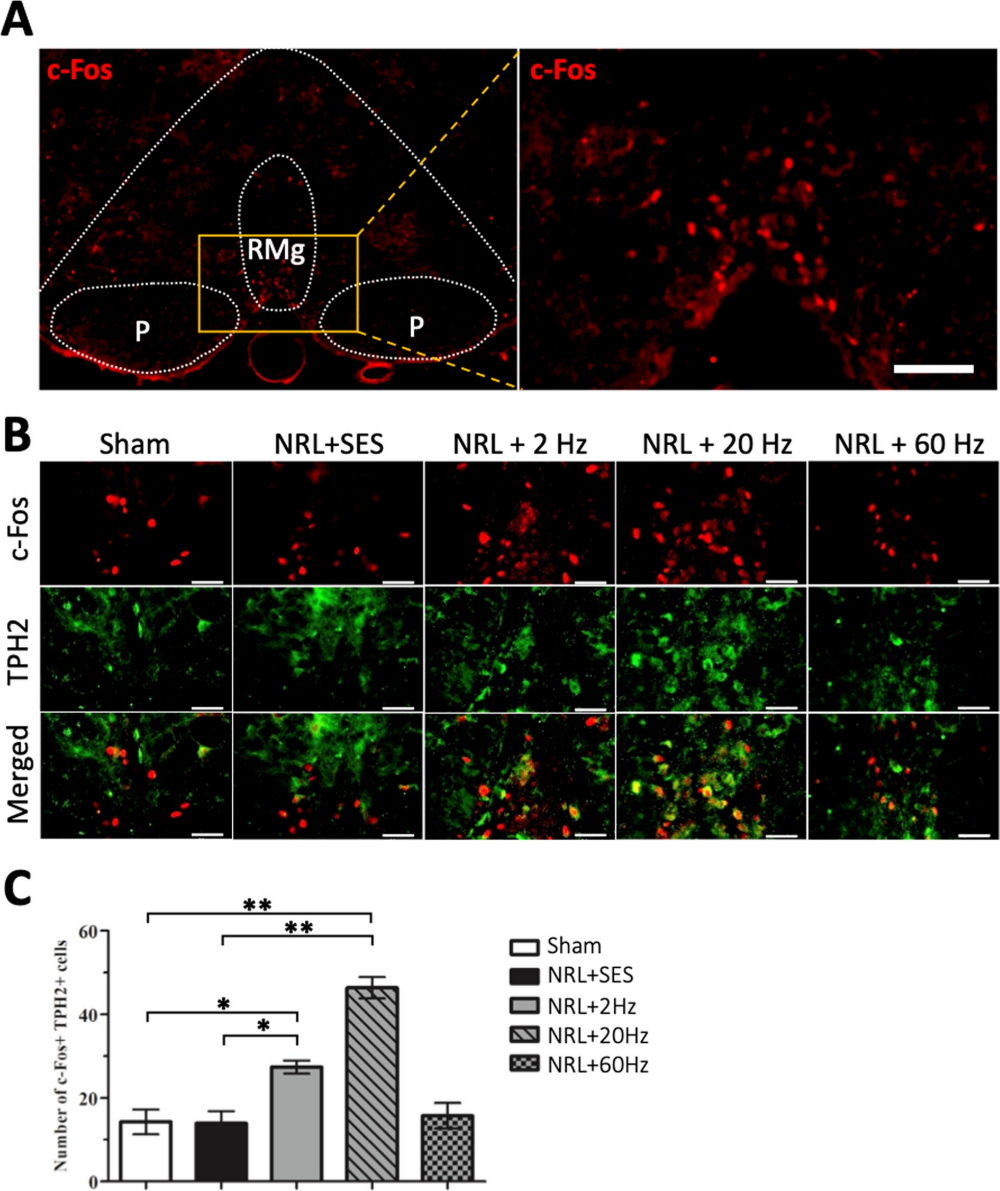
TPH2+ neuronal activity in the RVM following SNS at various frequencies compared to Sham stimulation in L5 NRL rats on PID1. Transverse sections of rostral brainstem were obtained from NRL rats on PID1 (**a**) (*N* = 3). Magnification of the RMg (A, right panel). Double-immunofluorescence staining of the serotoninergic neuron marker TPH2 (green) and c-fos (red) (**b**). Quantification of the number of TPH2/c-fos co-localized cells (**c**). Scale bar = 50 μm. Data are expressed as mean ± SD.*p < 0.05, **p < 0.01

To investigate the potential involvement of additional pain modulatory pathways, we analyzed the activity of the periaqueductal gray matter–rostral ventromedial medulla (PAG–RVM) descending pain modulation pathway. Figure 7 shows the immunofluorescence staining results of frozen RVM sections on PID 1. Colocalization of c-fos and TPH2 indicated the presence of activated serotoninergic neurons (Fig. 7b). Quantification of c-fos+/TPH2+ cells in the raphe magnus (RMg) nucleus revealed a 1.9-fold and 3.2-fold increase of c-fos + /TPH2+ cells following 2-Hz and 20-Hz SNS, respectively, compared to the Sham group (Fig. 7c). The changes in c-fos + /TPH2+ signals in the NRL + SES and NRL + 60 Hz groups were not significant compared to the Sham group.

## Discussion

A detailed characterization of the effect of SNS on NP models will increase our understanding of the mechanism of PNS to help achieve optimal pain relief. Moreover, research focusing on acute NP conditions may help broaden the range of clinical applications of PNS to benefit more patients in need. The main contribution of this study is demonstrating the analgesic efficacy of 2-Hz and 20-Hz SNS by showing alleviated pain behaviors and reduced SCDH hyperactivation in L5 NRL rats. Our results suggest that SNS plays an important role in pain modulation by reducing spinal cord inflammatory proteins and glial cell activation. Moreover, we showed an early increase in the activity of descending pain inhibition following 2-Hz and 20-Hz SNS, which may act in combination with the anti-inflammatory effect to achieve analgesia.

L5 NRL is a well-established rodent model of chronic NP that exhibits both pain and thermal hypersensitivity [25, 33]. In this study, the behavioral experiments validated the acute NP rat model of L5 NRL by showing that mechanical and thermal hypersensitivity occurred early on PID1 and were maintained until PID7. Although acute NP is increasingly clinically recognized [34], experimental models of acute NP are limited compared to those of chronic NP. Przewłocka et al. utilized crushing injury of the sciatic nerve as a model of acute NP and showed the development of allodynia on PID2; however, that study included no histological or biochemical analyses to supplement the pain behavioral test results [35]. In the current study, the behavioral phenotype of NP was supported by histological examination performed on both PID1 and PID7, revealing the hyperactivation of superficial laminar neurons in the SCDH, which are the major projection neurons in the spinothalamic tract.

In the present study, SNS was performed at three frequencies, namely 2 Hz, 20 Hz, and 60 Hz. Nerve stimulation at frequency in the range of 2–30 Hz is reported to exert an anti-inflammatory effect [36, 37]. Besides vagus nerve stimulation, which has already been approved for the treatment of systemic inflammatory cognitions [38], PNS on somatic nerves is reported to reduce inflammation [36, 39]. A study by Gürgen et al. demonstrated that perilesional transcutaneous electrical stimulation inhibited proinflammatory cytokines, including TNF-α, IL-1β, and IL-6, and improved wound healing [21]. Tu et al. proposed that, following PNS on sacral nerves, afferent signals were transmitted to the brainstem and that the efferent anti-inflammatory output is transmitted by the vagus nerve [40]**.** Despite recent reports of the use of SNS to treat chronic pain [41], whether SNS affects inflammation remains unclear. Notably, it has been reported that 60–100 Hz stimulation resulted in paresthesia of the innervated region and produced analgesic effects via the gate-control mechanism [42, 43]. As a result, a tingling sensation is not uncommon in patients receiving higher frequency stimulation [44].

In this study, the biochemical analyses were performed on PID1 and PID7. We sampled the tissues on PID1 to capture the analgesic effect that occurred early on PID1 in animal experiments. We investigated the morphologies on PID7 because it was reported that the first 7 days represent the developmental phase and the acute-to-chronic transition of NP [33]. Our results showed that, on PID1, all three frequencies of SNS alleviated the pain and thermal hypersensitivity induced by L5 NRL. However, only 2-Hz and 20-Hz SNS showed sustained analgesia on PID 3, 5, and 7. SNS with 60 Hz frequency only achieved analgesia on PID1 and the effect gradually declined. Although suppression of hyperactivated superficial SCDH neurons was observed at all frequencies on PID7, 60-Hz SNS had a lower efficacy. It is probable that the magnitude of suppression of hyperactivated superficial SCDH neurons resulting from 60-Hz SNS was insufficient to achieve analgesia. Additionally, it was also reported that the effect of high-frequency PNS may decline over time due to habituation [43], which might contribute to the different analgetic efficacy observed between 2 Hz, 20 Hz, and 60 Hz on PID7. Taken together, these results suggest that the mechanism through which 2-Hz, 20-Hz, and 60-Hz SNS achieve anti-nociception on PID1 may not be the same.

In light of the anti-inflammatory potential of SNS [21, 36], we hypothesized that modulation of CNS neuroinflammation may contribute to the observed differences in the effects of different SNS frequencies. Therefore, we investigated the neuroinflammatory response following L5 NRL and SNS. In the case of peripheral nerve injury, local inflammation at the injury site is followed by a proximal inflammatory response in the spinal cord [45]. Previous studies of peripheral nerve injury by Murphy et al. and Costigan et al. have revealed increased expression of proinflammatory cytokines, including TNFα, IL-1β, and IL-6, in the SCDH on PID7 after nerve injury and it is known that these cytokines are required for the development of pain [46, 47]. Consistent with those results, we demonstrated elevated expression of spinal cord IL-1β, IL-6, NF-κB, and TNF-α on day 7 after NRL. Our measurements of spinal cord inflammatory proteins showed that, although elevated expression of NF-κB and g TNF-α was not observed until PID7, increases in IL-1β and IL-6 expression occurred early on PID1. This result is compatible with a previous report that increased expression of IL-1β and IL-6 preceded TNF-α [48]. Overall, these findings suggest that IL-1β and IL-6 may be key initiators of neuroinflammation in NP and are thus potential therapeutic targets in the early stage of NP [45].

Next, because glial cells in the CNS are recognized as a major source of intrathecal IL-1β, IL-6, and TNFα [49], we investigated astrocyte proliferation and microglia activation in the SCDH following L5 NRL. Our results demonstrated astrocyte proliferation and microglia activation in the ipsilateral spinal cord on PID7. Neuronal–glial interactions play an important role in sensitizing and maintaining NP [50, 51]. In particular, Nam et al. showed that optogenetically induced spinal astrocyte activation governed the induction of pain hypersensitivity [9], while Gao et al. found that chemokine production in spinal cord astrocytes contributed to central sensitization of NP [52]. Furthermore, Ji et al. reported that spinal microglia are important contributors to NP development after nerve injury, and several studies have found that inhibiting microglial activation reduces hyperalgesia after nerve damage [53, 54]. Taken together, these findings identify SCDH astrocytes and microglia as potential therapeutic targets for NP. Relevant to this important point, we demonstrated that 2-Hz and 20-Hz SNS significantly ameliorated L5 NRL-induced astrocytic gliosis and microglial activation. The suppression of glial cell activation is also consistent with our finding of decreased expression of inflammatory proteins in the spinal cord after 2-Hz and 20-Hz SNS. Together, these results demonstrate the efficacy of 2 Hz and especially 20-Hz SNS in reducing spinal cord neuroinflammation in the L5 NRL NP rat model. Furthermore, our results suggested 20-Hz SNS may exert a better anti-inflammatory efficacy in the spinal cord.

Importantly, we noted that the analgesic effect of SNS in the behavioral experiments occurred early on PID1, whereas the anti-inflammatory effect of SNS was not significant until PID7. In light of the different results between the behavioral experiments and biochemical analysis, we hypothesized that other anti-nociceptive mechanisms may be involved. Given that the PAG–RVM descending pain modulation pathway is an early responder to pain and the RVM is activated 30 min after exposure to noxious stimuli [55], we hypothesized that descending pain modulation may contribute to the analgesic effect of SNS on PID1. Serotonin (5-HT) is the major neurotransmitter involved in pain modulation in the RVM and the descending serotonergic pathways mainly originate from the 5-HT rich RMg [56, 57]. Activation of this descending serotoninergic pathway has been shown to attenuate both mechanical and thermal hyperalgesia [56, 58]. As predicted, our results revealed that 2-Hz and 20-Hz SNS induced increased activity of serotoninergic neurons located at the RMg of the RVM on PID1. Early activation of the RVM descending pain modulation pathway could explain the reduced pain and thermal hypersensitivity behavioral test results on PID1.

Furthermore, our results showed that while the differences in the level of spinal cord IL-1β and IL-6 between L5 NRL rats with and without SNS were not significant on PID1, 2-Hz and 20-HZ SNS were able to attenuate the increased expression of spinal cord IL-1β and IL-6 on PID7. A reasonable explanation to this finding is the early resolution of neuroinflammation [20, 59]. Since neuroinflammation is a major contributor of central pain sensitization and central sensitization plays an important role in the formation of NP, early treatment with neuromodulatory techniques is proposed to have an additional advantage to block pain-induced maladaptive plasticity and neuroinflammation following neuronal injury, thereby preventing the acute-to-chronic pain transitioning [60, 61]. As a result, the therapeutic effect of early SNS treatment is likely a combination of increased descending pain inhibition, decreased and early-resolved neuroinflammation, and attenuated central pain sensitization [60].

Taken together, these results suggest that 2-Hz and 20-Hz SNS are effective in alleviating acute NP following L5 NRL in a rat model by suppressing SCDH neuroinflammation and facilitating descending pain inhibition. Comparisons between different stimulation frequencies suggest a neuromodulatory role of 2-Hz and 20-Hz SNS compared to the paresthesia effect of 60 Hz. Furthermore, our results demonstrate better efficacy of 20 Hz compared to 2 Hz for suppressing neuroinflammation and facilitating descending pain inhibition.

This study is subject to several limitations. Although we demonstrated the efficacy of SNS as a treatment for NP, the exact molecular mechanism of electrical peripheral neurostimulation has not been explored. Next, the timing of when SNS is performed may be less clinically applicable. Since this pilot study aimed to validate the potential therapeutic effect of SNS on NP and identify the optimal frequency, we performed SNS 2 h after L5 NRL. Future studies should investigate the optimal timing of SNS following injury to alleviate acute NP. Understanding the basis of these processes may provide insight to improve the currently available neurostimulation techniques to achieve better patient satisfaction. In-depth knowledge about the pathobiology of NP and the responses of neural–glial interactions to PNS may reveal additional therapeutic targets.

## Conclusions

In conclusion, our work has shown the analgetic effect of 2-Hz and 20-Hz SNS in an acute NP rodent model. We demonstrated regulation of inflammatory protein expression, astrocytic gliosis, and microglia activation following 2-Hz and 20-Hz SNS. During the early post-injury period, SNS might facilitate the descending pain modulatory pathway. Overall, our work has provided new insights into the cellular mechanisms of SNS and demonstrated its beneficial effect in acute NP.

## Supplementary Information

**Additional file 1.** Supplementary figure 1.

## Data Availability

The datasets used and/or analyzed during the current study are available from the corresponding author on reasonable request.

## Abbreviations

CNS: Central nervous system
NCKU: National Cheng Kung University
NP: Neuropathic pain
NRL: Nerve root ligation
PNS: Peripheral nerve stimulation
RMg: Raphe magnus nucleus
SCDH: Spinal cord dorsal horn
SCS: Spinal cord stimulation
SNS: Sciatic nerve stimulation
TENS: Transcutaneous electrical nerve stimulation
