# Supplementary figures and images for: Sciatic nerve stimulation alleviates acute neuropathic pain via modulation of neuroinflammation and descending pain inhibition in a rodent model
Source: J Neuroinflammation. 2022 Jun 15;19:153. doi: 10.1186/s12974-022-02513-y (PMC9199305; doi:10.1186/s12974-022-02513-y)

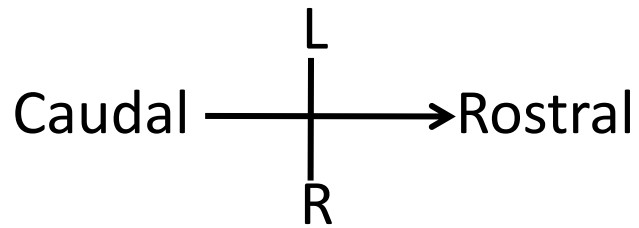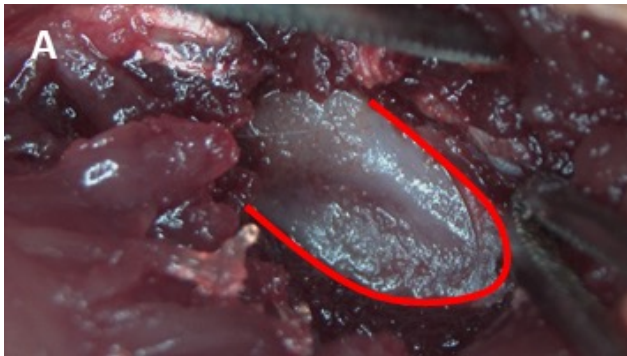

Exposure of L5  
transverse process

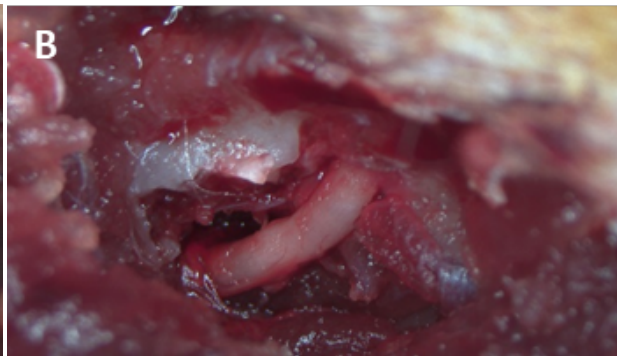

Exposure of L5  
nerve root

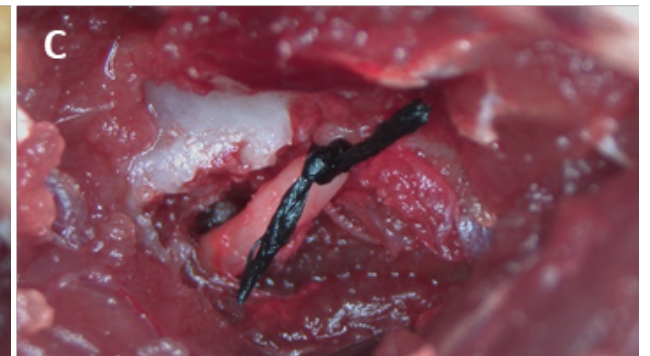

L5 nerve root  
ligation

Supplement: Supplementary file 1 — Additional file 1. Supplementary figure 1. [file 12974_2022_2513_MOESM1_ESM.pdf]
